# Methodological Challenges in Developmental Human Behavioral Epigenetics: Insights Into Study Design

**DOI:** 10.3389/fnbeh.2018.00286

**Published:** 2018-11-23

**Authors:** Livio Provenzi, Maddalena Brambilla, Renato Borgatti, Rosario Montirosso

**Affiliations:** ^1^Scientific Institute, IRCCS Eugenio Medea, 0-3 Center for the at-Risk Infant, Bosisio Parini, Italy; ^2^Scientific Institute, IRCCS Eugenio Medea, Neuropsychiatry and Neurorehabilitation Unit, Bosisio Parini, Italy

**Keywords:** behavioral epigenetics, developmental science, methodology, study design, DNA methylation

## Abstract

Developmental human behavioral epigenetics (DHBE) holds potential for contributing to better understanding of how early life exposures contribute to human developmental trajectories and to inform clinical practice and early interventions. Nonetheless, DHBE research to date is challenged by two major issues: (a) the frequent use of retrospective study designs; and (b) the major focus on epigenetic variations associated with early life adversities, rather than protective care exposures. In order for DHBE research to maintain its promises, these issues need to be addressed in a systematic way according to a careful methodological planning of study design. In this contribution, we provide pragmatic insights on methodological aspects that should be dealt with while designing DHBE studies. We propose different study designs for the retrospective and prospective investigation of both adversity- and care-related epigenetic variations. Examples from available scientific literature are provided to better describe the advantages and the limitations of each study design.

## Introduction

Behavioral epigenetics (BE; Lester et al., 2011) refers to the study of epigenetic mechanisms and variations in association with exposures to early environmental conditions and phenotypic developmental outcome. The emergence of BE as a relevant field of research is due to the pioneering work by Meaney and Szyf (2005) who reported on epigenetic variations (i.e., DNA methylation) observed in rats exposed to varying levels of caregiving quality within a normal range. Notably, exposures to early environmental conditions might include both the experience of adversities (e.g., prenatal and post-natal stress exposures; Hyman, 2009) as well as protective care conditions (e.g., high-quality caregiving; Curley and Champagne, 2016). On the one hand, rats exposed to low quality of caregiving (i.e., low frequency of linking and grooming and arch-back nursing) showed high levels of methylation at the exon 1F of a specific stress-related gene (i.e., glucocorticoid gene, *nr3c1*), which in turn was predictive of developmental outcomes including behavior regulation, social interactions and stress reactivity (Turecki and Meaney, 2016). On the other hand, when rats born by mothers rated as low-quality caregivers were subsequently cross-fostered to mothers rated as high-quality caregivers their *nr3c1* methylation patter was reversed to levels similar to those of rats born and raised by high-quality mothers and the detrimental effects on phenotype were no more observed (McGowan et al., 2011).

## The Emergence of Developmental Human Behavioral Epigenetics

The fascination arising from these original animal model research intrigued researchers involved in human developmental science to test whether such epigenetic mechanisms might be involved in the processes through which early experience is embedded into the phenotype (Lester et al., 2016). The gene X environment approach was providing insightful support to the notion that—at some point in life—our genetic predisposition and the environmental encounters might interact resulting in observable behaviors (Belsky and Pluess, 2012; Mileva-Seitz et al., 2016). Nonetheless, BE holds promise to reveal the biochemical processes through which this interaction actually occurs in a developmental framework (Lester et al., 2011; Conradt, 2017).

Developmental human behavioral epigenetics (DHBE) studies—i.e., the application of BE to the study of human development—rapidly emerged in scientific literature. To date, this research has revealed that early experiences of prenatal stress (Oberlander et al., 2008; Devlin et al., 2010; Sosnowski et al., 2018), maltreatment and abuse (Beach et al., 2010; Blaze et al., 2015; Tyrka et al., 2016), neonatal pain (Provenzi et al., 2015; Montirosso et al., 2016a; Fumagalli et al., 2018), socio-economic disadvantage (Essex et al., 2013; Swartz et al., 2017) and other adversities might lead to altered patterns of DNA methylation that contribute to an individual’s phenotype programming.

Nonetheless, very few DHBE studies to date focused on the epigenetic vestiges of protective care environmental conditions. For example, a retrospective study investigated the effect of maternal stroking on glucocorticoid receptor gene expression in infants that were exposure to maternal depression during pre and postnatal period (Murgatroyd et al., 2015). In this study, infants who were previously exposed to postnatal maternal depression showed a decrease of methylation after maternal stroking at 5 weeks of life. Consistent with this result, in a prospective study, the methylation correlates of physical maternal contact were measured in 4-to-5-year-old infants: those exposed to more frequent affectionate contact in the first 5 days of life showed greater methylation during preschool age (Moore et al., 2017).

## Challenges in DHBE Research

The misbalance between adversity- and care-focused BE research in human subjects is particularly evident. Different reasons might be invoked for such a predominant interest in adversity-related epigenetic variations in HBE. First, findings from animal models BE were more easily translated into study designs in human developmental science aimed at understanding the biological effects of early-in-life exposures to less-than-optimal caregiving contexts (Blaze et al., 2015). Second, the study of care-related epigenetic variations in animals can be relatively easy to set up with experimental manipulation, which is not the case of human subjects (Lester et al., 2011). Third, in humans protective and care interventions are delivered concurrently or—more often—after the occurrence of stressful, traumatic or adverse conditions and the timing of adversities and protective care is only partially predictable. As a result, in the absence of a conjoint and integrated DHBE research on both adverse and protective exposures, limited implications can be derived from this field of research that might have clear and insightful implications for clinical practice.

Moreover, the majority of the available DHBE research is characterized by retrospective study designs. The prevalence of correlational research adopting retrospective investigations might be at least partially related to the fact that when Meaney and Szyf’s findings on rat’s caregiving and DNA methylation variations began to be published, researchers involved in human studies started to assess DNA methylation by applying epigenetic investigation *post hoc* to ongoing cohorts. Another reason to set up more retrospective and cross-sectional studies than prospective and longitudinal ones in DHBE might be due to limitations of experimental manipulation in human subjects. As stressful or adverse conditions might not be “administered” to infants and children as well as to adult human subjects for ethical reasons, in most of the cases epigenetic mechanisms can be investigated only at *post hoc*. Notably, this limitation mostly applies to DHBE focused on early adversities, whereas it is less valid for BE research on the epigenetic correlates of protective care interventions in human subjects. Whereas retrospective and cross-sectional designs allow to set up DHBE research to reveal potential associations between environmental exposures and variations in DNA methylation, to date the lack of prospective and longitudinal research leads to reduced capacity of developing causal interpretations and theoretical modeling.

## The Present Contribution

In the present study, we provide an in-depth discussion of different study designs in DHBE, we describe in details the advantages and limitations of different methodological architectures and we explore how different designs can be affected or solve major challenges in human BE research. When available, examples from research to date are also reported to better highlight the benefits and the challenges inherent to each specific methodological approach.

## Retrospective and Cross-Sectional Study Designs

### Type 1: Concurrent Assessment of Epigenetics and Outcomes

#### Description

**Figure 1:**
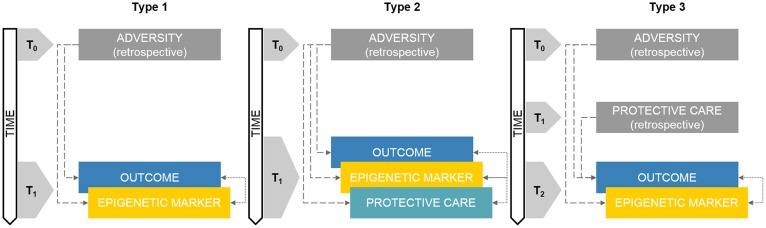
Schematic representations of retrospective developmental human behavioral epigenetics (DHBE) study designs. Note. Type 1: concurrent assessment of epigenetics and outcomes; Type 2: concurrent assessment of epigenetics, care exposures and outcomes assessment; Type 3: retrospective exposure to adversity/care and concurrent assessment of epigenetics and outcomes. Gray boxes reflect retrospective measurements. Double-headed arrows reflect correlational nature of the association. Colored boxes reflect non-retrospective assessments. The annotation t_n_ refers to progressive time-points in the research study design.

As reported in Figure 1 (Type 1), this kind of study design implies the presence of an adverse exposure which has occurred before the study began (T_0_) and that can be only measured or accounted for through retrospective investigations such as self-report, medical charts, socio-demographic records and other proxies. The core focus of this study design is on the assessment of both epigenetic variations and the identified outcome(s) at T_1_.

#### Example

In a recent article, Cicchetti and Handley (2017) reported on the retrospective investigation of the link between maltreatment, glucocorticoid receptor gene (i.e., *NR3C1*) methylation and several outcomes (i.e., emotional lability, self-control, behavioral problems, depressive symptoms) in a cohort of more than 500 school-aged children. Maltreatment information was obtained through the consultation of healthcare services records, classified in subtypes (i.e., neglect, emotional maltreatment, physical and sexual abuse) and characterized in terms of severity, age at exposure and chronicity. Cross-sectional assessment of *NR3C1* methylation and outcomes occurred in a single research session. Children with history of early-onset maltreatment presented significant greater methylation of the *NR3C1* gene when compared to non-maltreated counterparts. The cumulative exposure to more than one maltreatment subtype and to chronic maltreatment was significantly linked with *NR3C1* hyper-methylation, which, in turn, was associated with greater emotional lability, lower self-control, more externalizing behavioral problems and depressive symptoms.

#### Advantages

The cross-sectional nature of the study is generally associated with a less expensive research in terms of human resources. Moreover, researchers do not have to wait for environmental conditions to develop in time in order to obtain assays of epigenetic biomarkers and outcomes, as the stressful conditions are already occurred prior to the study onset. Additionally, no experimental manipulations of environmental conditions are required, thus partially limiting the potentials of ethical issues for at-risk or fragile human subjects with a history of adverse exposures. This type of retrospective studies is well suited to investigate potential associations which have never been previously tested, providing preliminary evidence of putative significant correlations and co-variations between specific epigenetic markers and developmental outcomes.

#### Limitations

As the information on adversity exposure and stressful conditions can only be obtained indirectly, many efforts should be devoted in controlling as many confounding sources as possible. Nonetheless, the absence of direct account of stressful exposures (i.e., the actual duration or accurate observation of specific stress conditions) is a major flaw of this kind of DHBE study design. Additionally, the kind of depicted relationship among variables (i.e., retrospective adversity indexes, epigenetic markers and outcomes) can only be correlational and no assumption can be proposed on the direction of the effect between epigenetic marker and outcome measures. Finally, in absence of two sequential measures of epigenetic markers, there is no possibility to obtain a variability index assessing the actual effect of adversity on the quantitative change in the selected epigenetic marker.

### Type 2: Concurrent Assessment of Epigenetics, Care Exposures and Outcomes Assessment

#### Description

In this type of study design, adversity exposure already occurred before the subjects were enrolled in the research project (T_0_) whereas epigenetic markers, protective care conditions and outcomes are obtained within the same cross-sectional research session (T_1_; see Figure 1, Type 2). Compared to Type 1 study design, this methodological setting adds the integrated investigation of adversity and protective care exposures in a single DHBE research program.

#### Example

Conradt et al. (2016) investigated the association among the exposure to maternal post-natal depression, DNA methylation of *NR3C1* and placental 11*β*-hydroxysteroid dehydrogenase Type 2 gene (i.e., *11β-HSD-2*), salivary cortisol reactivity to socio-emotional stress (i.e., the five-episode Face-to-Face Still-Face; Provenzi et al., 2016) and maternal sensitivity in 128 4-month-old infants. Retrospective self-report measure of maternal depressive symptoms was obtained through questionnaire administration. Infants’ DNA methylation, hypothalamic-pituitary-adrenal (HPA) axis regulation in response to the FFSF procedure and maternal sensitivity were all measured in the same research session at 4-month-age. As such, this study design is cross-sectional and retrospective, but has the merit of providing a preliminary account of the concurrent associations of adversity (i.e., maternal depression) and protective care (i.e., maternal sensitivity) with infants’ DNA methylation and developmental outcome (i.e., stress regulation). Indeed, the authors showed that infants from depressed mothers who exhibited high levels of sensitivity during the face-to-face interaction occurring just before the still-face episode of the FFSF procedure had lower *NR3C1* and *11β-HSD-2* methylation levels compared to counterparts from depressed mothers with lower sensitivity ratings. Consistent with previous animal model research (Curley et al., 2011), this study suggests that the quality of maternal caregiving behavior might be a significant protective buffer in the face of early adversity exposure in human infants.

#### Advantages

The limited impact on human resources and the possibility to obtain in relative short amount of time information on a whole cohort of subjects also applies to Type 2 study design. Also, limited manipulation of experimental conditions is allowed and care dimensions usually include naturally available protective factors such as socio-economical conditions or normal variations in parental care. These studies also have the advantage of testing uninvestigated hypotheses on the potential buffering or mediating role of protective care factors on the association among early adversities, epigenetic markers and selected developmental outcomes.

#### Limitations

Two major flaws affect this kind of study design: first, the indirect and retrospective account of adversities, as in Type 1 research; second, the concurrent assessment of protective factors and outcomes, which do not allow the interpretation of the direction of potentially significant associations. As such, while Type 2 research increases the complexity and provides more information compared to Type 1 study design, this DHBE research is still characterized as correlational and more careful interpretations of the study results should be done. Still, only epigenetic markers—but not variability indexes associated with adverse and protective exposures—are usually obtained in Type 2 research.

### Type 3: Retrospective Exposure to Adversity/Care and Concurrent Assessment of Epigenetics and Outcomes

#### Description

In this kind of study design, three time-points are considered (Figure 1, Type 3). Adversity exposure occurred in T_0_, usually well before (e.g., previous generation) the actual assessments included in the research program. Protective factors occurred in T_1_, which is still antecedent to the study onset, but subsequent to adversity in T_0_. The research assessments occur in T_2_ and usually include at least one of the following measurements: (a) epigenetic marker (e.g., DNA methylation) and (b) developmental outcome (e.g., health-related indexes). As consequence, in this kind of studies, both adverse and protective exposures are measured indirectly through retrospective investigations, whereas epigenetic and outcome measurements occur usually concurrently in one research session.

#### Example

The project Ice Storm is a well-suited example of the Type 3 study design (Cao-Lei et al., 2018). In this research, adversity (T_0_) consists in the well-known ice storm disaster that occurred in Quebec in 1998 and which offers a unique condition to assess intergenerational effects of early stressful conditions (King and Laplante, 2005). Ice storm exposure was quantified through retrospective questionnaire measuring the “objective hardship” of the exposure, including threat, loss, scope and change. Protective factor (T_1_) consisted in the maternal capacity of cognitive reappraisal coded as “negative” *vs*. “neutral and positive” meaning making and coping strategies. At the time of assessment (T_2_), enrolled subjects from the generation subsequent to that exposed to the 1998 ice storm averaged 13.6 years old and they were assessed for C-peptide secretion in blood T cells and for DNA methylation at selected genes associated with risk of Type 1 and 2 diabetes. The main merit of this study is the possibility to test for independent effects of adversity (i.e., objective hardship) and protection (i.e., maternal cognitive reappraisal) as well as for mediation models including both environmental exposures and epigenetic markers (i.e., DNA methylation at target sites within selected genes). Indeed, the authors showed that a direct marginal effect emerged only for objective hardship, whereas DNA methylation of diabetes-related genes significantly mediated the effects of objective hardship (positive mediation) and cognitive reappraisal (negative mediation) on risk for diabetes (i.e., C-peptide secretion) in children.

#### Advantages

Although both adversities and protective care exposures are quantified at *post hoc*, this study design allow researchers to build a more complex model of the interactions as well as of the individual and joint contributions that environmental conditions exert on infants’ epigenetic regulation and developmental outcomes. More specifically, the presence of a temporal sequence that organizes stress and protection conditions in the past of enrolled individuals adds to the possibility to develop a theoretical framework in which the buffering effect of protective factors can be appreciated.

#### Limitations

In the light of advantages listed above, major limitations still apply to this complex, yet retrospective study design. First, in the absence of an initial epigenetic assay at T_0_—or even before—there is no way researchers can observe and report on epigenetic regulation and variations in time and on the effects of environmental conditions for bad and for good on this time-related change. Second, despite a temporal sequence is hypothesized in the past occurrence of adversity and protective factors, careful interpretation of findings still applies to this kind of research, as it cannot be entirely excluded that other protective factors might have intervened concurrently or even before T_0_. Other flaws previously reported in relation to the retrospective assessment of adversity and protection (see Type 1 and Type 2 above) still apply to the present DHBE research program and only correlational interpretations of the relationship between epigenetic markers and developmental outcomes are sustained.

## Prospective and Longitudinal Study Designs

### Type 4: Prospective Assessment of Adversity-Related Epigenetic Variations on Longitudinal Outcomes

#### Description

**Figure 2:**
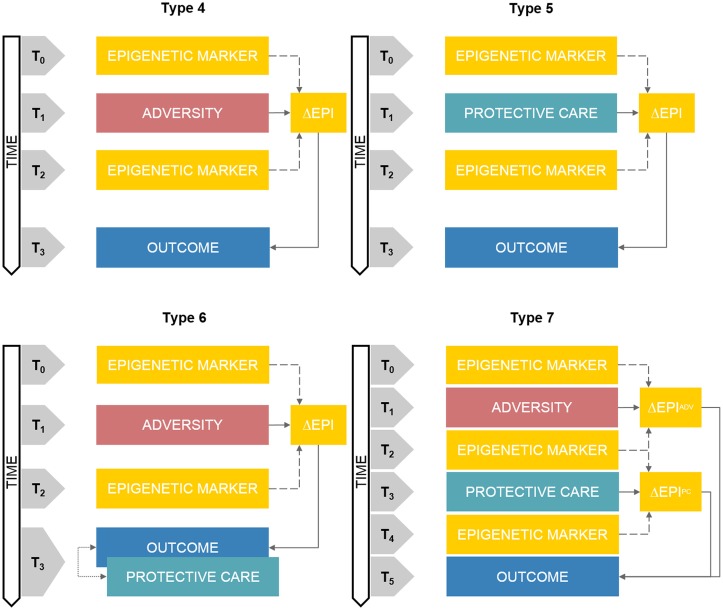
Schematic representations of prospective DHBE study designs. Note. Type 4: prospective assessment of adversity-related epigenetic variations on longitudinal outcomes. Type 5: clinical trials of care-related epigenetic variations on longitudinal outcomes. Type 6: prospective assessment of adversity-related epigenetic variations on longitudinal outcomes with concurrent exposure to protective care. Type 7: prospective and sequential assessment of adversity- and care-related epigenetic variations on longitudinal outcomes. ΔEPI box, Epigenetic variation; ΔE^ADV^ box, Adversity-related epigenetic variation; ΔE^PC^ box, Protective care-related epigenetic variation. Double-headed arrows reflect correlational associations. The annotation t_n_ refers to progressive time-points in the research study design.

The key feature of this research architecture is the presence of at least two assessments of epigenetic markers at two different time points (see Figure 2, Type 4). Between the first (T_0_) and the second (T_2_) epigenetic assay, a prospective quantification of an adverse-related exposure is carried on (T_1_). A longitudinal assessment of a developmental outcome (T_3_) can be also included at least once after the second epigenetic assessment.

#### Example

Recent research applying epigenetic research principles and methodology to the study of early stress exposure in preterm infants during the hospitalization in the Neonatal Intensive Care Unit (NICU; Montirosso and Provenzi, 2015) represents a prototypic research context to describe Type 3 study design (Provenzi et al., 2017a). In this research program, epigenetic markers are obtained at birth (T_0_) and at NICU discharge (T_2_), whereas during the NICU stay (T_1_) specific variables related to stress exposures (e.g., pain-inducing skin-breaking procedures, mechanical ventilation, maternal separation) are quantified in a prospective way, usually on a day-by-day basis. Outcomes (e.g., socio-emotional stress regulation; Montirosso et al., 2016a) are measured at specific post-discharge time-points (T_3_). In a recent article from this emerging field of research (Fumagalli et al., 2018), structural equation modeling was applied to assess the mediation role of epigenetic variation (e.g., difference in the methylation levels of the serotonin transporter gene, *SLC6A4*, from birth to discharge) on the association between NICU-related stress and anterior temporal lobes volumes at 12-month corrected age in preterm infants.

#### Advantages

The presence of two epigenetic measurements in different time-points allow researchers to move from epigenetic markers to epigenetic variations. Ratios and/or differential indexes of this variation in time can be obtained and are warranted to be tested in association with the target environmental exposure. Second, the kind of association that can be observed between adversity and epigenetic variation might also be characterized in terms of direction and hypothesis on the effect of adversity on epigenetic variation can be advanced. Third, a long-term chain of action-reaction sequences can be tested in complex models including all the variables, from the first epigenetic assay at T_0_ to the outcome assessment(s) at T_3_. More specifically, a prospective evaluation of the mediation of epigenetic variation on the association between adversity and outcome can be tested consistent with the temporal sequence in which environmental exposures and assessments occurred.

#### Limitations

Limitations of retrospective designs (Type 1–3) do not apply to prospective studies. Nonetheless, specific issues and challenges should be highlighted. First, these studies are much more expensive in terms of research procedures and human resources. Second, the longitudinal nature of the study exposes the research plan to moderate-to-high risk of sample attrition, as subjects enrolled at T_0_ might not be available for the subsequent research sessions from T_1_ to T_n_ due to many different reasons. Third, mainly for ethical reasons, contrary to what happens in animal model research, the adversity exposure cannot be experimentally manipulated or induced. As such, human populations who are naturally exposed to less-than-optimal developmental conditions featuring stress exposure are the best eligible subjects for this kind of research. In other words, this study design should be considered as quasi-experimental and careful interpretation of the direction of the effects is recommended.

### Type 5: Clinical Trials of Care-Related Epigenetic Variations on Longitudinal Outcomes

#### Description

This study design differs from Type 4 basically because of the type of environmental exposure which is assessed, namely protective care conditions (see Figure 2, Type 5). Indeed, between the first (T_0_) and the second (T_2_) epigenetic assay, some kind of protective care intervention is promoted and offered to at least one sub-group in the study sample (T_1_). A longitudinal assessment of a developmental outcome (T_3_) can be also included at least once after the second epigenetic assessment.

#### Example

Research on the effects of brief psychotherapy interventions appear to be among the best examples of the Type 5 prospective DHBE study design. Roberts and collaborators (Roberts et al., 2014) assessed *SLC6A4* methylation before (T_0_) and after (T_2_) the exposure to a cognitive-behavior therapy (T_1_) in children with anxiety disorder. Post-treatment effects were measured in terms of a reduction of anxiety symptoms occurring at 6-month follow-up (T_3_). The findings suggested that *SLC6A4* methylation partially explained the difference in the reduced anxiety symptomatology between children who improved and those who did not after the psychotherapy.

#### Advantages

The inclusion of a protective care intervention instead of an adverse condition in T_1_ is not only a mere difference in terms of the object of investigation. Rather, the possibility to set up an intervention implies much more control for researchers and this kind of study design actually can be described as a clinical trial, with variable degrees of control and randomization. As such, this is probably one of the most robust methodological architectures for DHBE research. At the same time, this field of research holds potentials for the most direct implications and insights to advance evidence-based clinical practice and to inform early healthcare interventions for at-risk infants and children.

#### Limitations

The focus on prospective exposure and measurement of protective care interventions requires the setup of an integrated multi-professional research team and an accurate standardization of the intervention. At greater degrees of methodological control of confounders, more than one group of subjects is needed and infants/children included in the groups need to be matched for a number of socio-demographic, clinical and task-relevant variables. At lower degrees of control and randomization, only one group can be included, but less robust interpretations are supported.

### Type 6: Prospective Assessment of Adversity-Related Epigenetic Variations on Longitudinal Outcomes With Concurrent Exposure to Protective Care

#### Description

Many variants of Type 4 and 5 study designs can be developed to include an integrated assessment of both adversity and protective care exposures into a DHBE research project. One of this variant presents the inclusion of a concurrent assessment of a specific protective exposure at T_3_—together with the target outcome measurement—increasing the complexity of Type 4 study design (see Figure 2, Type 6).

#### Example

Preterm birth and its consequences in terms of early hospitalization and developmental outcomes represent an elite condition for designing Type 6 research projects. Using this kind of methodological architecture, Provenzi and colleagues (Provenzi et al., 2017b) assessed the moderation role of maternal sensitive interactive behavior during a face-to-face interaction (T_3_) with full-term and preterm infants on the association between NICU-related (T_1_) variations in *SLC6A4* methylation (T_0_ and T_2_) and infants’ behavioral stress regulation (i.e., negative emotionality; T_3_) at 3 months of age (corrected for prematurity). Notably, this study suggested that a significant moderation emerged only for full-term infants, in which mothers rated as highly sensitive appeared to protect their infants from the positive association between high *SLC6A4* methylation and heightened negative emotionality. By converse, no significant moderation emerged for preterm infants, indirectly supporting the need of DHBE research focused on an integrated investigation of NICU-related adversity and specific early interventions occurring well before outcomes’ assessment (see Type 7 below).

#### Advantages

Type 6 study design combine the benefits of a prospective and longitudinal research project reported above (see Types 4 and 5) with the opportunity to appreciate the role of protective factors as post-adversity buffering mechanisms on developmental outcomes. This methodology is highly indicated and potentially insightful for areas of clinically applied research in which high individual variability is documented as a consequence of early stress exposure. In other words, this study can reveal the biological pathways that contribute to the fact that some individuals seem to cope adequately and develop well in the face of a developmental history of adversities, whereas others do not.

#### Limitations

Despite the many advantages of this study design, two major flaws should be highlighted. First, this kind of research can be applied only to those human developmental conditions that are inherently distressful and in which infants or children are naturally exposed to stressful environments (see Type 4 limitations). Second, the concurrent evaluation of outcomes and care variables do not allow researchers to assess the independent role of protective factors as contributors to epigenetic regulation.

### Type 7: Prospective and Sequential Assessment of Adversity- and Care-Related Epigenetic Variations on Longitudinal Outcomes

#### Description

This is probably the most complex study design within the landscape of DHBE research, as at least seven time-points are included and complex relationship among exposures, epigenetic variations and outcomes are addressed (see Figure 2, Type 7). Epigenetic assessments occur before (T_0_) and after (T_2_) an adverse event exposure (T_1_). After that, protective care intervention is promoted and quantified prospectively (T_3_) and a third epigenetic assessment occurs after that (T_4_). At least one post-intervention time-point is included for follow-up evaluation of the developmental outcomes (T_5_). Unfortunately, to the best of our knowledge, there is no evidence of such research projects in the available DHBE literature.

#### Advantages

The sequential order of exposures and epigenetic as well as outcome assessments allows researchers to develop specific and methodologically sound hypotheses that can tested and falsified with a modular approach. These can include simple hypotheses regarding two or three subsequent time-points as well as more complex modeling hypotheses including the integrated assessment of different environmental exposures, epigenetic variations and outcomes. The assessment of the competitive, additive or independent effects of adversity and protective care on the same targets of epigenetic variations and developmental outcomes is a specific plus of this study design which cannot be pursued with other methodological architectures.

#### Limitations

Whereas this study design maximizes the benefits at the methodological level, it should also be clear that resource costs, the risk for longitudinal sample attrition and the involvement of a multi-professional team of researchers and clinician imposes relevant challenges so that risks and potential solutions need to be carefully planned before and revised during the research project. At the same time, these research projects can be only applied to naturally occurring adversities for which protective care interventions are already available and provided some levels of evidence for the specific selected developmental outcomes. From this point of view, research on preterm behavioral epigenetics (PBE) still represents an elite field of applied research for DHBE (Maddalena, 2013; Provenzi and Barello, 2015). Nonetheless, it should be noted that a simplified version of Type 7 study design can be also suggested, in which a mixed retrospective assessment of adversity exposure and a prospective evaluation of care-related epigenetic variation together their effects on developmental outcomes of are featured. In this mixed design, T_0-to-2_ would be collapsed into one single retrospective assessment, reducing the interpretive potentials of the model, but allowing its application to many different adverse conditions and reducing the risk of longitudinal sample attrition.

## Discussion

DHBE is one of the most promising and rapidly growing areas of developmental science in human psychobiological research field. These studies are well-recognized as holding promises of advancing our comprehension of the biological mechanisms underlying the association between environmental exposures and the developmental phenotype (Szyf et al., 2008; Griffiths and Hunter, 2014). Additionally, the growing body of information on the epigenetic targets and markers of early environmental exposures—for bad and for good—is going to inform smarter and effective evidence-based clinical practice for infants and children who present genetic and/or environmental risk factors (Bianco-Miotto et al., 2017; Murgatroyd and Spengler, 2011; Notterman and Mitchell, 2015). Nonetheless, DHBE presents specific challenges and issues, when it comes to make methodological choices and produce a robust study design. In the present contribution, we have proposed several different prototypical and didactically organized study designs to cope with these challenges.

One major distinction among different methodological approaches regards the use of retrospective or prospective measurements of environmental exposures, either adversities or protective conditions. Retrospective approaches clearly have the advantage of being applicable to a number of adverse conditions which can be faced by human beings and that cannot be experimentally induced or manipulated (e.g., abuse and maltreatment; Beach et al., 2013; Parent et al., 2017). Moreover, retrospective study designs represent a compromise solution that balances the amount of resources needed to the research project and the strength of methodological procedures and robustness of findings. Indeed, retrospective designs are usually correlational and no reliable interpretation can be supported for what pertains predictive effects and the direction of potential significant associations. As such, these study designs are better suited for exploratory research projects and to test innovative and previously uninvestigated hypotheses that hold the potential to pave the way for more research in the field.

By converse, prospective approaches usually implies more complex study designs which increase the robustness of findings’ interpretation, allow the formulation of more specific hypotheses on the direction of predictive associations and effects. At the same time, they imply new challenges such as the high risk of sample attrition and subjects’ loss and the careful planning of multiple research sessions within specific time frames. Prospective study designs are highly indicated when environmental conditions—for bad and for good—can be longitudinally followed-up, monitored and quantified and/or when greater opportunities for experimental manipulation and control is granted. For instance, this is the case of PBE research (Provenzi et al., 2018a), in which the early exposure to adverse conditions (e.g., NICU-related pain-inducing procedures) and care interventions (e.g., skin-to-skin contact support) are expected and can be easily measured on a daily basis for a relatively long period (Provenzi et al., 2015; Montirosso et al., 2016a). Prospective DHBE studies are particularly well suited for the investigation of specific hypotheses regarding the predictive effects of adversities and protective care interventions on target epigenetic regulation.

Another major difference between retrospective and prospective study designs regards the kind of epigenetic indexes that can be obtained. On the one hand, retrospective and cross-sectional study designs provide the context for the assay of punctual epigenetic markers, which can be considered as potential endophenotype associated with specific environmental exposures (Radley et al., 2011). On the other hand, prospective research allows the measurements of epigenetic variations from pre- to post-exposure, which further increases the possibility to provide stronger interpretations on the direction of the effects and supports the development of more complex mapping of the reciprocal interactions among environmental conditions, epigenetic regulation and developmental outcomes. In other words, while retrospective approaches to DHBE provide photograph-like accounts of the relationships among environmental exposures, epigenetic markers and individuals’ phenotype, prospective study designs provide the methodological requisites to depict a movie-like systematic landscape of such relationships.

In light of considerations reported above, we would like to highlight specific implications for future studies in DHBE research field. First, a more explicit account of the limitations of the adopted study design and their advantages and disadvantages for direct and indirect implications and interpretations should be provided to readers in DHBE studies. As previously suggested, the “seductive allure” (Miller, 2010) of BE research and its application to human developmental science might pave the way for misinterpretations in the absence of scientifically sound supervised and guided readership (Richardson et al., 2014; Provenzi and Montirosso, 2015). Second, as more and more DHBE research get published in the scientific community, there is a need to provide systematic accounts of this rapidly growing body of evidence, to weight them and to highlight the gray areas of under-investigated yet clinically relevant areas of BE applications for human developmental research. For example, the imbalance between DHBE research focused on adversities and on protective care exposures is evident and more efforts should be devoted to care-related DHBE studies in the years to come. Third, in the face of the described challenges inherent to the application of BE research principles to the study of human development, more direct and frequent contacts between animal model and human epigenetics research should be pursued. Investing in this multi-professional communities of BE researchers is warranted to provide a more systematic account and robust rationale for the applied and basic evidence that can support our improved knowledge of developmental mechanisms and our capacity to provide smarter and more effective early interventions in at-risk conditions. Additionally, it should be highlighted that the use of peripheral tissues for measuring DNA methylation variations is the only available proxy in DHBE, whereas epigenetic research on animal models rely on central nervous system tissues. Recent research reported on partial correspondence in DNA methylation measured from peripheral blood and saliva (Thompson et al., 2013; Staunstrup et al., 2017), but still methylation status appears to be tissue- (Smith et al., 2015; Forest et al., 2018) and gene-specific (Di Sante et al., 2018). Also, Walton et al. (2016) reported only 7.9% significant correlation between methylation measures in blood and brain tissue obtained in post-mortem patients: despite this proportion was greater than predicted by chance, it is still a small proportion. As such, a direct translation of findings from animal model studies to humans is discouraged, in absence of multiple evidence and cross-tissue reports (Lester et al., 2011). Finally, as previous DHBE research mainly focused on DNA methylation, in this article we limited our discussion and examples to this epigenetic mechanism. Nonetheless, it should be noted that DNA methylation can have direct and indirect effects of the transcription machinery (Curradi et al., 2002). Moreover, other epigenetic mechanisms have been documented in associations with early adversities, at least in animal models (e.g., histone regulation, Cittaro et al., 2016; non-coding RNA, Daskalakis et al., 2018; telomere length regulation, Provenzi et al., 2018b) and might interact with each other in contributing to establish chromatin availability to transcription and gene silencing (O’Leary et al., 2017).

In sum, it should be highlighted that when it comes to apply BE principles to human developmental science, there is no “best approach” and that the balance between benefits and challenges should be considered for each specific research project based on a conjoint evaluation of researchers’ aims, objects of investigation and available resources. Although non-exhaustive, this article provides a wide overview of study designs and offers methodological support and guidelines to researchers who are interested in integrating BE methods and investigation in their developmental science research. A well-designed research program is key to develop robust methodology for future DHBE studies that can be beneficial for increasing and deepening our knowledge of developmental mechanisms and inform early interventions for at-risk infants and children.

## Author Contributions

LP conceived the idea. LP and MB drafted the manuscript. RM and RB critically revised the manuscript for important intellectual content. All authors gave final approval of the final version and are accountable for this work. The corresponding author attests that all listed authors met authorship criteria and that no others meeting the criteria have been omitted.

## Conflict of Interest Statement

The authors declare that the research was conducted in the absence of any commercial or financial relationships that could be construed as a potential conflict of interest.
